# Quantum Modeling: A Bridge between the Pumping and Signaling Functions of Na/K-ATPase

**DOI:** 10.3390/ijms19082347

**Published:** 2018-08-09

**Authors:** Weiguang Wang, Joseph I. Shapiro

**Affiliations:** The Joan C. Edwards School of Medicine, Marshall University, 1600 Medical Center Drive, Suite 3408, Huntington, WV 25701, USA; wang68@marshall.edu

**Keywords:** Na/K-ATPase, Markov chain, master equation, Src, oxidant stress, reactive oxygen species, aging

## Abstract

Although the signaling function of Na/K-ATPase has been studied for decades, the chasm between the pumping function and the signaling function of Na/K-ATPase is still an open issue. This article explores the relationship between ion pumping and signaling with attention to the amplification of oxidants through this signaling function. We specifically consider the Na/K-ATPase with respect to its signaling function as a superposition of different states described for its pumping function. We then examine how alterations in the relative amounts of these states could alter signaling through the Src-EGFR-ROS pathway. Using assumptions based on some experimental observations published by our laboratories and others, we develop some predictions regarding cellular oxidant stress.

## 1. Introduction

**Figure 1:**
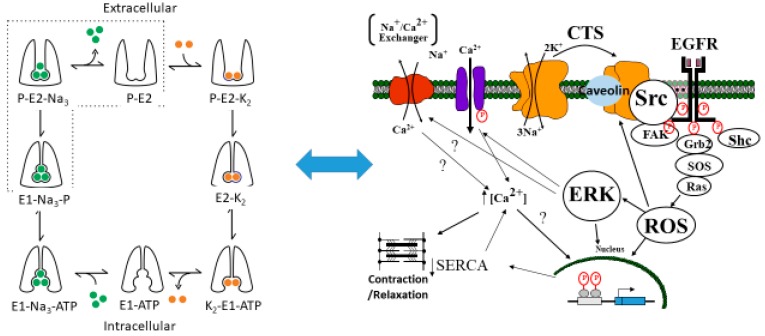
The proposed relationship between the Post-Albers model for the Na/K-ATPase (**left panel**) and signal transduction through caveolar Na/K-ATPase-Src-EGFR cascade. The Post-Albers model shows putative transitions between E1 and E2 states, as well as ion trafficking. Note that, according to this model, the Na/K-ATPase in the E1 state releases two K molecules in the cytosol, picks up three Na molecules, and is phosphorylated. It then coverts to the E2 state where the Na molecules are released into the extracellular space and two K molecules are picked up from this extracellular space, ultimately to be released into the cytoplasm as the cycle repeats. The **right panel** shows signal transduction through Src, the epidermal growth factor receptor (EGFR), and other signaling partners, including src homology 2-containing protein (Shc), growth factor receptor 2 (Grb2), and focal adhesion kinase (FAK), leading to son of sevenless (SOS) and Ras activation, which ultimately results in ROS generation which feeds back to the Na/K-ATPase. This putative schema discussed in several reviews [10,11,12].

The Na/K-ATPase is a P-type ATPase discovered in the 1950s by Skou [1]. It is arguably one of the most important proteins in animal biology. It has been known since Skou’s original discovery to play a key role in ion transport (hence its alternate name, “sodium pump”), and the conformational changes which occur during the solute pumping have been elucidated specifically by Post and Albers, whose names grace the model most often used to describe this ion transport by this protein [2,3]. However, in addition to this ion transport function, studies from the Xie/Shapiro laboratories also showed that the Na/K-ATPase also plays an essential role in cell signal transduction and reactive oxygen species (ROS) regulation [4,5,6]. It has been postulated that this signaling function is related to a scaffolding function of the Na/K-ATPase controlling the activity of the rous sarcoma gene product (Src) kinase [7,8,9]. This is illustrated in Figure 1. 

Although this postulate has attracted some controversy [13], we would argue that the effectiveness of therapeutics developed entirely on the basis of this scaffolding function demonstrate its validity [14,15,16,17]. For the purpose of this exploration into modeling, we propose the following axioms based on this postulated scaffolding function.

1. The Na/K-ATPase α1 subunit inhibits associated Src kinase while in the E1 state. This has been suggested by modeling studies that demonstrate a number of poses by which a peptide in the N domain of the α1 subunit of the Na/K-ATPase interacts with the kinase domain of Src while in the E1 state, but an absence of such poses for the E2 state [18].
2. Activation of Src kinase leads to transactivation of the epithelial growth factor receptor (EGFR) and downstream generation of reactive oxygen species.

As mentioned above, axiom 1 has evidence in [18,19], whereas axiom 2 is supported by extensive data published in [5,6,19,20,21,22,23]. 

Although for many years, focus has been on the effect that a class of molecules referred to as cardiotonic steroids (CTS) signal through the Na/K-ATPase, more recent evaluation demonstrates that the Na/K-ATPase can function as a feed-forward amplifier for oxidants [14,15,16,18,19]. Based on these data, we propose the following axiom:

3. ROS both result from the activation of the Na/K-ATPase-Src-EGFR signal cascade and can initiate the previously discussed activation by oxidation of the Na/K-ATPase which, in turn, decreases the tonic inhibition of associated Src Kinase. We further assume (candidly without much proof at present) that the oxidation of the Na/K-ATPase results in an increase in time than the Na/K-ATPase spends in the E2 state. The data for ouabain or other CTS increasing time in the E2 state are excellent. What is also clear is that either the binding of CTS or exposure to exogenous oxidants leads to the identical oxidation of amino acids in the A domain detailed in [18]. Alterations in the lipid environment of the Na/K-ATPase induced by oxidants is another possibility that must be studied further.

To further incorporate understandings gleaned from extensive experiments probing the functional relationship between the scaffolding function of the Na/K-ATPase and cell biology, we suggest that:

4. The level of oxidant stress determines the rate of endocytosis of the Na/K-ATPase complex from the plasma membrane which results in a net disinhibition of membrane associated Src. This axiom is supported by extensive studies examining endocytosis in porcine kidney cell line (LLC-PK1) cells and rat proximal tubule cells which both respond to either oxidants or CTS with endocytosis of the basolateral Na/K-ATPase and redistribution of the apical sodium proton antiporter isoform 3 (NHE3) in an oxidant-dependent manner [21,23,24,25].

From these assumptions or axioms, we will attempt to model the relationship between signaling through the Na/K-ATPase and redox state within the cell. Recent studies show that the normal Na/K-ATPase has a rapid velocity of conformational change during its pumping function. For instance, some studies show that purified Na/K-ATPase hydrolyzed ATP at 1000–1500 µmol/mg protein/h [26]. As a result of this rapid changes in conformation, characteristics of the Na/K-ATPase, especially binding coefficients, will not hold constant. In fact, we have modelled this with respect to the scaffolding function—tonic inhibition of Src kinase based largely on molecular modeling presented in [18]. 

## 2. The Markov Chain Model

First, we assume that confrontational change of a single Na/K-ATPase unit is simply (and sometimes incorrectly) independent from adjacent conformations during its turnover process, therefore, its turnover process could be depicted by the Markov chain model [27]. Obviously, at the equilibrium state, this single Na/K-ATPase unit will essentially represent a superposition of a number of conformations which can be described as follows:

(1) $$\left\{X_{n},n\in T\}(T=0,1,2,\ldots \right)$$

Assume that after a very short time interval Δt, this single Na/K-ATPase unit will randomly switch its conformation from one to another, which only depends on its previous conformation. Obviously, this probability could be described as:

(2) $$P\{X_{n+1}=i_{n+1}|X_{n}=i_{n}\}$$

The term *P* means the probability of this single Na/K-ATPase unit changes its conformation from conformation *i* to conformation *i* + 1, the time interval between *n* and *n +* 1 is Δt. Similarly, between every possible conformation *i* and *j* (*i*, *j* ∈ *n*), a one-step transform probability could be defined as:

(3) $$p_{ij}(n)=P\{X_{n+1}=j|X_{n}=i\}$$

Which means that for every given conformation *i* and *j*, there is always a corresponding probability *p_ij_*. If we restrict ourselves to the 4 major states in the Post-Albers model:

(4) $$\begin{array}{ccccc} & E1 & E1P & E2P & E2\\ E1 & p_{11} & p_{12} & p_{13} & p_{14}\\ E1P & p_{21} & p_{22} & p_{23} & p_{24}\\ E2P & p_{31} & p_{32} & p_{33} & p_{34}\\ E2 & p_{41} & p_{42} & p_{34} & p_{44} \end{array}\;$$

However, it is certainly possible (in fact likely) that there are any number of “hidden” states (possibly representing those associated with binding different numbers of Na or K molecules). If those “hidden states” need to be considered, the one-step transition matrix could be written as:

(5) $$\begin{array}{cccccccccc} & E1 & \ldots & E1P & \ldots & E2P & \ldots & E2 & \ldots & E_{1j}\\ \vdots & \vdots & \ddots & \ddots & \ddots & \ddots & \ddots & \ddots & \ddots & \vdots \\ E1 & p_{11} & \ldots & p_{12} & \ldots & p_{13} & \ldots & p_{14} & \ldots & p_{1j}\\ \vdots & \vdots & \ddots & \ddots & \ddots & \ddots & \ddots & \ddots & \ddots & \vdots \\ E1P & p_{21} & \ldots & p_{22} & \ldots & p_{23} & \ldots & p_{24} & \ldots & p_{2j}\\ \vdots & \vdots & \ddots & \ddots & \ddots & \ddots & \ddots & \ddots & \ddots & \vdots \\ E2P & p_{31} & \ldots & p_{32} & \ldots & p_{33} & \ldots & p_{34} & \ldots & p_{3j}\\ \vdots & \vdots & \ddots & \ddots & \ddots & \ddots & \ddots & \ddots & \ddots & \vdots \\ E2 & p_{41} & \ldots & p_{42} & \ldots & p_{43} & \ldots & p_{44} & \ldots & \vdots \\ \vdots & \vdots & \ddots & \ddots & \ddots & \ddots & \ddots & \ddots & \ddots & p_{ij} \end{array}$$

When the single Na/K-ATPase reaches the equilibrium state (*n*→∞), this Markov process could be defined as a stationary distribution as a result of *p_ij_*(*n*) is independent from *n*:

(6) $$\pi =(\pi_{j},j\in I),\pi_{j}\leq 0$$

(7) $$\underset{n\to \infty }{\lim}X^{\left(n\right)}=\underset{n\to \infty }{\lim}PX^{\left(n-1\right)}=\pi ,$$

(8) $$\pi_{j}=\sum_{j\in I}\pi_{i}p_{ij},\sum_{j\in I}\pi_{j}=1$$

As a result of motor proteins, assume every *p_ij_* has a corresponding *k_ij_* between conformation *i* and *j*. Thus, consider the probability density *p_ij_*(*t*) denoted by the master equation:

(9) $$\frac{d}{dt}p_{ij}\left(t\right)=\underset{j\neq i}{\sum }\left[k_{ij}^{+}\left(t\right)p_{j}\left(t\right)-k_{ij}^{-}\left(t\right)p_{i}\left(t\right)\right]$$

The transition rate $k_{ij}^{+}$ and $k_{ij}^{-}$ mean the conformation change rate of Na/K-ATPase from conformation *i* to *j* and *j* to *i*, respectively. According to the laws of thermodynamics, the number of reactions per unit time *(J^NESS^*) and the amount of heat dissipated into the environment per unit time (*e_p_*) could be written as [28]:

(10) $$J^{NESS}=\frac{k_{1}^{+}k_{2}^{+}k_{3}^{+}\ldots k_{n}^{+}-k_{1}^{-}k_{2}^{-}k_{3}^{-}\ldots k_{ij}^{-}}{\left\{k_{1}^{+}k_{2}^{+}+k_{1}^{-}k_{2}^{1}\ldots \ldots +k_{n-1}^{+}k_{ij}^{-}\right\}}$$

(11) $$e_{p}=J^{NESS}ln\left(\frac{k_{1}^{+}k_{2}^{+}\ldots k_{ij}^{+}}{k_{1}^{-}k_{2}^{-}\ldots k_{ij}^{-}}\right)$$

Although nontrivial, this calculation suggests that a measurement of *e_p_* would allow for some estimation regarding these transitions. Developing strategies to minimize and/or maximize the probability of different states could allow for calculation, at least on the level of an ensemble for these *k* values. Unfortunately, it is likely (as we proposed) that there are different pools of Na/K-ATPase which have different rates of transitions. Hence, while the above calculations might be possible to describe one of these pools, we would need to separately consider those *e_p_* and associated *k* values associated with the pumping and signaling portions of the Na/K-ATPase. Again, there are biochemical and molecular biological methods that are available to selectively deplete the signaling portion of the Na/K-ATPase allowing for at least the possibility of performing these measurements.

## 3. Superposition of States

**Figure 2:**
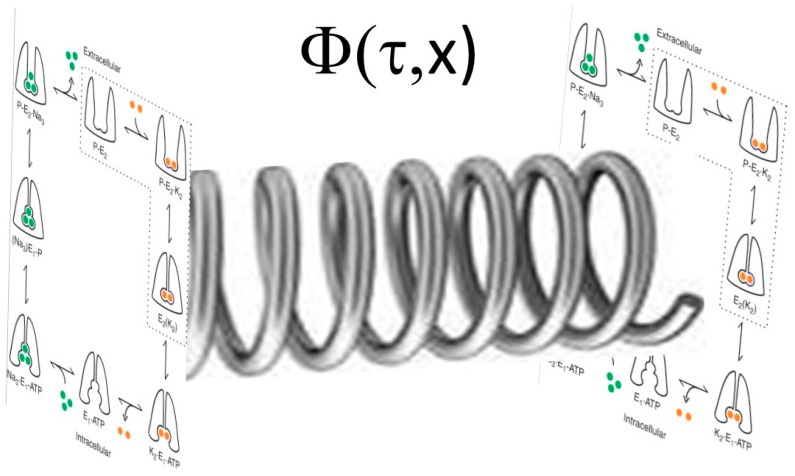
Post-Albers model of Na/K-ATPase shown as a wave function in the complex plane with transitions between the E1 and E2 states with different phosphorylation steps shown.

A related, but more simplistic, approach would be as follows: if we imagine in the complex plane, one could contemplate a wave function to describe the different states of the Na/K-ATPase, as shown in Figure 2. 

Arbitrarily, we could consider the “real” values to represent transitions between E1 and E2, whereas the “complex” values would represent the degree of phosphorylation. If we further assume that there are both spatial and frequency separation of the “signaling” Na/K-ATPase (i.e., that Na/K-ATPase present in caveolae and complexed with caveolin 1, Src, and other signaling partners) and “pumping” pump (that Na/K-ATPase is not associated with caveolin 1, Src, and other signaling proteins), one can imagine the pump as a plane wave with two different spatial domains and two different frequencies as shown below (the R program used to generate these figures is shown in Appendix A).

**Figure 3:**
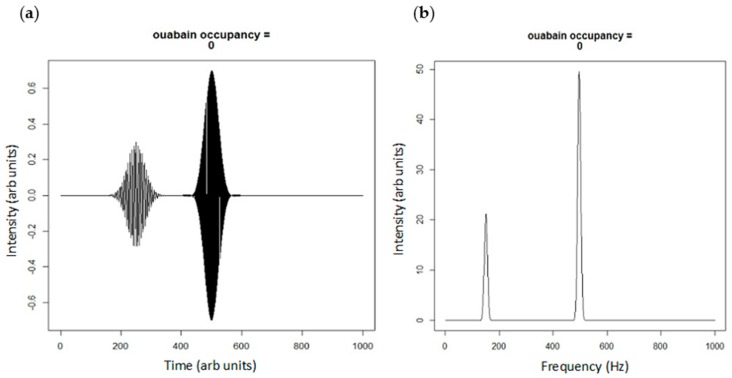
(**a**) Real projection of superposition of spatially (500 vs. 250 arb units) and frequency (500 vs. 150 Hz) distinct portions of Na/K-ATPase; and (**b**) the power spectrum of the Fourier transform of the plane waves in Figure 3a showing portions of signaling and pumping Na/K-ATPase. The peak at 150 Hz could be considered a net Src inhibition according to our model.

**Figure 4:**
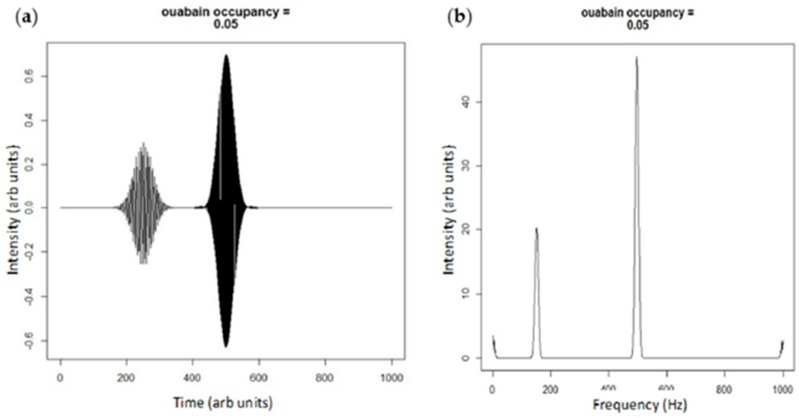
(**a**) Plane wave simulation of ouabain binding shifting the E2:E1 state by 5%; and (**b**) the Fourier transform of the adjacent simulation. Note that the height of the smaller peak centered at 180 Hz is diminished compared to Figure 3b representing net Src activation.

Although we have previously described a pool of “non-pumping” pumps to describe the signaling portion of the Na/K-ATPase [29], it is far more likely (and consistent with our other observations) that the signaling portion of the Na/K-ATPase cycles through different conformations more slowly and is, thus, much less relevant to ion transport that the non-signaling pool. While it is a gross oversimplification to consider residence within caveolae to define the signaling vs. pumping pump, it does appear that the signaling pump does associate with Src and caveolin [8]. However, to illustrate the difference (Figure 3 and Figure 4), we will continue to use both frequency and space to separate these two pools. If we look at ouabain as a pharmacological tool to shift more Na/K-ATPase into the E2 state (ultimately dis-inhibiting Src kinase), the wave function simulations might look something like Figure 4.

## 4. Extension of the Markov Chain to Define the Oxidant Amplification Loop

If we accept the axioms stated above, it is clear that Src kinase activity is a master-controller of subsequent signal transduction and ROS generation. As ROS can also initiate this sequence through altering Na/K-ATPase conformation and dis-inhibiting Src kinase, feed forward amplification results [18]. 

Although a number of additional assumptions must be made to construct the model, we chose to attempt to look at ROS regulation from this system. The assumptions necessary for this simple system of differential equations include (detailed R program shown in Appendix A):

- The Na/K-ATPase has a basal synthesis rate.
- ROS shift the Na/K-ATPase into the E2 state (as do CTS).
- There is a basal rate of ROS production from other sources.
- There is a link between Src phosphorylation and ROS production (oversimplifying the cascade, which we believe involves transactivation of the EGFR and other steps).
- There is a detoxification rate of ROS which is proportional to ROS concentration (an assumption that is at least partially true, based on the kinetics of superoxide dismutase and catalase).
- There is a relationship coupling ROS concentration to rates of endocytosis of the Na/K-ATPase [30].
- There is a decay rate of phosphorylated Src which is proportional to the amount of phosphorylated Src.

For the purpose of the model, we will further assume that the cells in question have approximately 2 M Na/K-ATPase units on the surface with about ½ of Src regulated by this pool of the “signaling” Na/K-ATPase. This is similar to what we have seen with LLC-PK1 cells. We further assume (and model) that the usual proportion of the Na/K-ATPase in the E1 state is about 50% with units of ROS being entirely arbitrary (vida infra). This is consistent with the observed co-dependence of the velocity of Na/K-ATPase on both intracellular Na and extracellular K concentrations [31]. 

**Figure 5:**
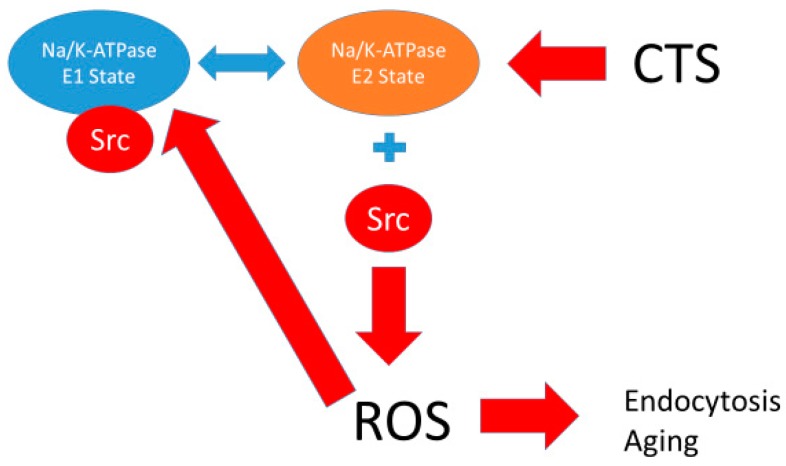
Schematic of Na/K-ATPase showing the release of Src with conversion into the E2 state (stimulated by ROS and CTS) with further generation of ROS inducing endocytosis and aging. See associated differential equations in Appendix A.

**Figure 6:**
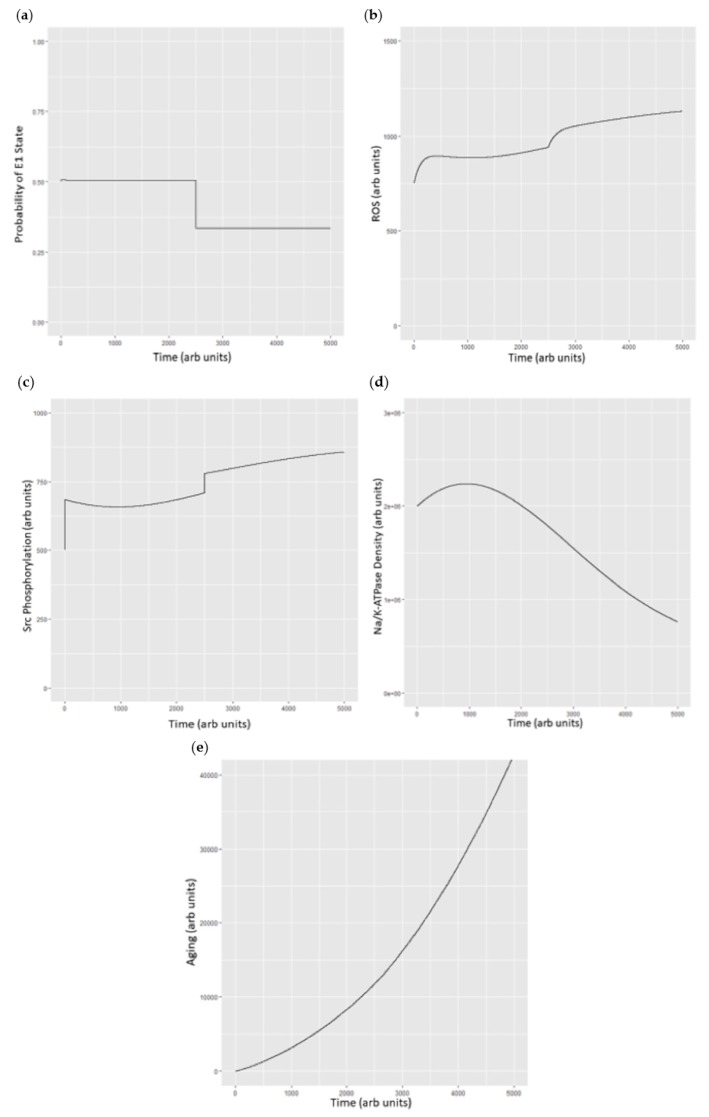
(**a**) Probability of the E1 state under basal conditions and then with the simulated addition of ouabain at 100 µM (approximately 10% of the IC_50_) at time = 2500; (**b**) simulated changes in ROS under basal conditions and after addition of simulated ouabain at time = 2500. Units of ROS are arbitrary; (**c**) Src phosphorylation as total phosphorylated Src vs. time with conditions described above. Src concentrations are arbitrary beyond the understanding that ½ of the Src pool is under control of the Na/K-ATPase and ½ is not; (**d**) plasma Na/K-ATPase under basal conditions and following addition of ouabain which stimulates endocytosis (at t = 2500); and (**e**) cell aging as a function of ROS with ROS generated through the Na/K-ATPase oxidant amplification loop and Na/K-ATPase endocytosis set to be approximately 1000 times as efficient and inducing “aging”, as we have recently described [32].

The ROS concentrations are more difficult. What we know from mammalian biology is that there are a number of interdependent processes leading to ROS generation including the Na/K-ATPase oxidant amplification loop [32]. Simplistically, we know that oxidization of sulfhydryl groups occurs with oxidant stress, whereas reduction occurs because of the generation of reducing equivalents, largely (although not entirely) through the hexose monophosphate shunt. Rate constants to make the model “work” were arrived at with an iterative approach, seeking stability prior to the “shift” if E1/E2 states achieved a specific time point (see Appendix A). A high level schematic of this process which we subject to simulation is shown in Figure 5. The simulations are shown in Figure 6. 

What we see in experimental manipulations is that shifting a portion of the Na/K-ATPase into the E2 state with ouabain results in a surge in ROS and endocytosis of the Na/K-ATPase [6]. 

The models discussed above are clearly simplistic and need validation of assumptions used in their construction. However, it is fair to say that the model (as it was constructed to simulated this) does give roughly similar changes in Src phosphorylation and ROS generation (as well as cellular aging) as that seen in experimental systems [5,6,18,24,29,32]. In addition, there are several additional points which can be drawn:

1. The Na/K-ATPase can be viewed as existing in a superposition of states which relate to each other as a Markov chain.
2. The Na/K-ATPase interacts with Src and its other signaling partners using principles also seen with Markov chains. This allows modeling to be developed using a system of ordinary differential equations.
3. As seen in biological systems, oxidant stress results from the shift in the Na/K-ATPase to the E2 state, which then becomes ineffective in inhibiting Src [18]. This leads to a cascade generating oxidants which further stimulates the Na/K-ATPase oxidant amplification loop (feed forward amplification) as well as oxidant injury consistent with aging.

As we consider these points, we realize that none of these insights require the modeling efforts made in this paper. Moreover, there are a large number of assumptions used in the creation of the models that are very difficult to validate with experimental data. We should also emphasize that we do not consider any dynamic changes in Na or K gradients that may develop during the course of simulations as we did not feel this was fruitful for acute perturbations. However, we do believe that we can use these models and further refinements of these models to make predictions in different cells and tissues, and ultimately expand our understanding of this pathway, which is the topic of this symposium.

## Appendix A. R code for Oxidant Stress/Aging Simulation

#NKA signaling simulation

# load the libraries

library(deSolve)

#set up function to alter E1 to E2 transition

duration=5000

ko=function(t,duration){

  if(t<duration/2){

  return(0)

  }else{

  return(1e-3)

  }

}

# set up parameters

parms=c(GNKA=800 #rate of NKA synthesis

   ,K1=9.5e-4 #rate constant shift to E2 state

   ,K_1=1e-3 #rate constant shift to E1 state

   ,K2=3e-8 #effect of ROS on E1 E2 states

   ,KSR=1e-2 #link between Src and ROS

   ,GROS=5e-1 #basal rate of ROS synthesis

   ,K3=1.2e-3 #dephosporylation rate of Src

   ,K21=8e-3 #decay of ROS

   ,K4=-2.5e-10 #conversion of ROS into endocytosis

   ,K5=0

   ,K6=10 #link between endocytosis and ROS and aging"

   ,K7=.001 #link between ROS and aging

   ,K8=1.1e-6 #phosphorylation of Src

   )

state=c(N=2E6,T=1E6, p=5e2, R=750, E=-0.00015, PE1=0.5,A=0)

#set up differential equations

SIMNKA=function(t,state,parms){

  with(as.list(c(state,parms)),{

  dN=GNKA+E*N

  dT=K5*T

  dp=K8*((1-PE1)*N/4 + T-N/4)*(T-p)-K3*p*T

  dR=GROS+KSR*p-K21*R

  dE=K4*R

  dPE1=-(K2*R+K1+ko(t,duration))*(PE1*N)+K_1*(1-PE1)*N

  dA=(K6*-1*E+K7)*R

  list(c(dN,dT,dp,dR,dE,dPE1,dA))

  })

}

# solve differential equations with a solver

times=seq(from=0,to=duration, by=0.1)

out=lsoda(y=state,times=times, func=SIMNKA,parms=parms)

# plot results

library(ggplot2)

out=as.data.frame(out)

windows()

p=ggplot(out)+geom_line(aes(x=out[,1],y=out[,2]))+coord_cartesian(xlim=c(0,5000),ylim=c(0,3e6))+ggtitle("plasmalemmal Na/K-ATPase")

plot(p)

windows()

p=ggplot(out)+geom_line(aes(x=out[,1],y=out[,4]))+coord_cartesian(xlim=c(0,5000),ylim=c(0,1e3))+ggtitle("Src phosphorylation")

plot(p)

windows()

p=ggplot(out)+geom_line(aes(x=out[,1],y=out[,5]))+coord_cartesian(xlim=c(0,5000),ylim=c(0,1.5e3))+ggtitle("ROS")

plot(p)

windows()

p=ggplot(out)+geom_line(aes(x=out[,1],y=out[,7]))+coord_cartesian(xlim=c(0,5000),ylim=c(0,1))+ggtitle("Prob of E1 state")

plot(p)

windows()

p=ggplot(out)+geom_line(aes(x=out[,1],y=out[,8]))+coord_cartesian(xlim=c(0,5000),ylim=c(0,40000))+ggtitle("Aging (arb units)")

plot(p)
